# Polymeric Coatings with Antimicrobial Activity: A Short Review

**DOI:** 10.3390/polym12112469

**Published:** 2020-10-24

**Authors:** Ana C. Pinho, Ana P. Piedade

**Affiliations:** Department of Mechanical Engineering, CEMMPRE, University of Coimbra, 3030-788 Coimbra, Portugal; acspinho5@gmail.com

**Keywords:** antimicrobial polymeric coatings, surface topography, surface chemistry, monolithic coatings, polymer-based composites

## Abstract

The actual situation of microorganisms resistant to antibiotics and pandemics caused by a virus makes research in the area of antimicrobial and antiviral materials and surfaces more urgent than ever. Several strategies can be pursued to attain such properties using different classes of materials. This review focuses on polymeric materials that are applied as coatings onto pre-existing components/parts mainly to inhibit microbial activity, but polymer surfaces with biocidal properties can be reported. Among the several approaches that can be done when addressing polymeric coatings, this review will be divided in two: antimicrobial activities due to the topographic cues, and one based on the chemistry of the surface. Some future perspectives on this topic will be given together with the conclusions of the literature survey.

## 1. Introduction

There are several reasons why microorganisms, particularly bacteria, developed resistance to antibiotics: incorrect medical prescription [1], incompletion of appropriate antimicrobial therapy by the patient [2], overuse [3], extensive agricultural use [4], availability of few new antibiotics [5], and the overuse of antibacterial products for hygienic or cleaning purposes [6]. The fact is that, independently of the reason, researchers have devoted strenuous efforts in developing materials that can present antimicrobial and/or biocidal activity to overcome the stated problem. A wide range of medical components, more prone to bacterial infections and more serious consequences, are based on polymeric materials mainly due to their chemical stability, good mechanical properties, manufacturing versatility, and lower cost.

Polymers are very versatile materials and, since long ago, have been modified to present additional properties/characteristics for different applications [7,8,9]. Nevertheless, by themselves or after some chemical modification, polymers can present antimicrobial properties, as described in many excellent papers, such as the one in ref [10]. However, often polymeric materials do not present the appropriate bulk properties that enable them to be applied in components/parts for structural engineering applications and invasive medical devices that require similar mechanical solicitations. In those situations, the polymer, monolithic, or composite, is used as a coating to confer to the surfaces of other materials the desired antimicrobial properties. This review focuses on this aspect, which explores both the topographic and chemistry cues used as strategies to prevent microbial adhesion and proliferation.

## 2. Polymeric Coatings

Several techniques can be used to produce coatings that are deposited onto a specific surface. The methodologies can be divided according to several approaches, considering if the deposition occurs at ambient conditions or under vacuum, according to the few given examples. Within the techniques used at ambient conditions, the simplest one usually involves the evaporation of the solute from a polymeric solution placed onto the desired substrate. Sometimes, the blade methodology can be used to control the amount of polymer onto the substrate [11]. This methodology is generally used for flat substrates, whereas for the coating of complex three-dimensional substrates is usually achieved through dip coating [12] and spray [13] methods. Within the methodologies used at ambient conditions, spin coating produces polymeric coatings with high quality [14].

Among the techniques that are used under vacuum conditions for the deposition of polymeric coatings, two of the most cited in the literature are the chemical vapor deposition (CVD) [15] and the physical vapor deposition (PVD) [16]. The latest is considered one of the cleanest technologies for the deposition of coatings of any class material, as it does not involve any chemical or hazardous materials [17,18,19].

However, within this review, the emphasis will not be given to the technique but to the resulting polymeric coatings and their characterization, particularly their antimicrobial performance. This aim will be presented in the next sub-sections.

### 2.1. Topographic Cues

A significant percentage of the available literature that studies the influence of the topographic cues onto the antimicrobial activity of polymers is not related to the roughness surface parameters of polymeric coatings. Instead, they describe the effect of engineered micro- and nano-features produced onto the surface of bulk polymers. Such is the case of surface engineered PEEK (polyetheretherketone) with cone/pillar-like micro/nano-arrays that mimic insect wings found to have antimicrobial activity. However, this behavior depended on the overall surface topography and dimensions of the surface structures [20]. Similar to this approach, several micro pattern architectures inspired in nature have been used to produce antimicrobial surfaces. Patterns, such as pits, pillars, ribs, channels, and ridges, are some examples engineered onto bulk surfaces [21].

If the only cue to be considered is the topographic one, only very few published articles use this type of approach. Most of the literature survey results in manuscripts were the influence of the topography is studied together with chemical modifications produced in the surfaces. One of the most recent works reports the plasma polymerization of the antibacterial polyterpenol onto several titanium substrates with different roughness at the nanoscale [22]. Nevertheless, the authors attribute the difference in the antibacterial properties, towards *Staphylococcus aureus* (*S. aureus*) and *Pseudomonas aeruginosa (P. aeruginosa),* to a heterogeneous chemical distribution of some chemical functional groups induced by the different topography. As reported in the paper, the topographic cues are not directly responsible for the more or less antimicrobial effect but induce different chemical surfaces responsible for the antibacterial effect.

**Figure 1:**
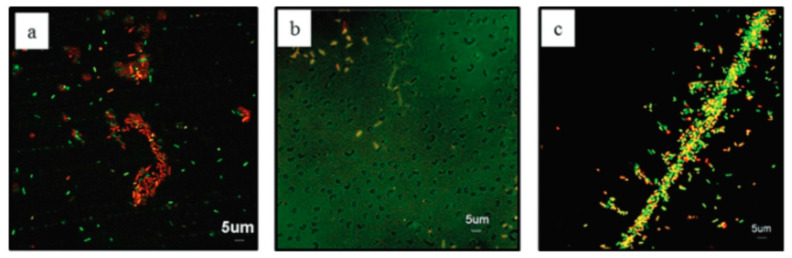
Confocal Laser Scanning Microscopy images of *E. coli* attached to glass (**a**) to ppOct (**b**) and to ppAAc (**c**) after 18 h of incubation at 37 °C. Bacteria were stained using LIVE/DEAD^®^ BacLight^TM^ Bacterial Viability kit. Green cells are considered alive, and red cells are considered dead. Reprinted with permission from [23]. Copyright 2015, American Vacuum Society.

One other work highlighting the difficulty of establishing only the effect of topography onto the bacterial attachment was published by Pegalajar-Jurado and collaborators [23]. The authors used colloidal lithography and plasma polymerization, onto glass substrates, to produce periodic nanotopographies with controllable surface chemistry. The attachment of *Escherichia coli (E. coli)* onto carboxyl and hydrocarbon plasma polymer films both on flat and array surfaces was studied (Figure 1). The authors concluded that surface chemistry played a critical role in bacterial attachment, whereas the exclusive effect of surface nanotopography was almost impossible to access.

The influence of the topography on *P. aeruginosa* attachment was evaluated onto a polyimide biomedical film [24]. The authors induced micrometric periodic arrays of lines with 1, 2 and 10 µm period using Direct Laser Interference Patterning (DLIP). The authors find that the only the highest period was not able to inhibit bacterial adhesion.

However, the results cannot be evaluated only considering the topographic effect as the authors indicate that the DLIP processing also induces chemical changes in the surface of the polymer. Moreover, they attribute the observed degree of bacterial colonization to the different wettability of the surface, but they did not correct the measured contact angle values considering the different surface roughness. Therefore, once again no unequivocal conclusions regarding the topographic effect in the antimicrobial activity can be obtained.

**Figure 2:**
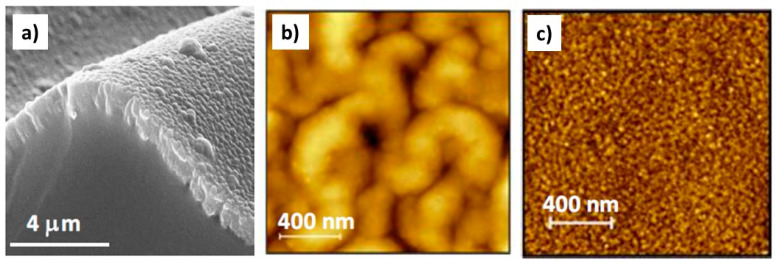
Different surface morphology and topography, evaluated by SEM and AFM, of a polyamide coating deposited onto PDMS (**a**,**b**) and Si (**c**) substrates. Reprinted from [25], published by MDPI.

In a recent work, a polymer coating was sputtered onto the surface of an elastomer, polydimethylsiloxane (PDMS), creating hierarchical micro- and nanowrinkled surfaces, and simultaneously onto Si wafers to produce nanorough surfaces [25] (Figure 2).

**Figure 3:**
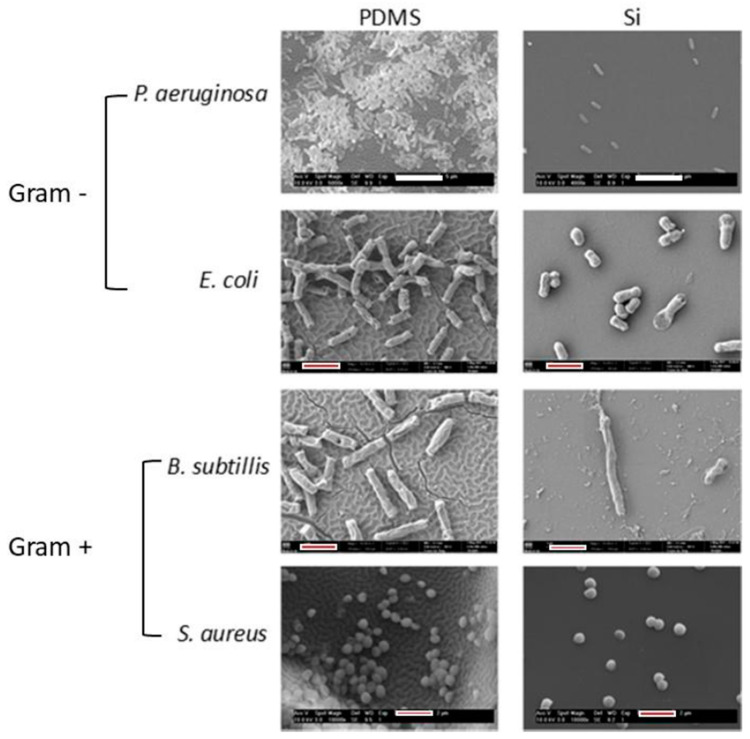
SEM micrographs of the polyamide thin film deposited onto PDMS and Si after the incubation with bacterial strains in solid medium (white bars = 5 μm; red & white bar = 2 μm). Reprinted from [25], published by MDPI.

The coating was obtained from a polyamide target to promote a surface with a chemical composition attractive for bacterial colonization to evaluate the true effect of topography. In this study, four bacterial strains were used: two Gram-negative *E. coli*, *P. aeruginosa*, and two Gram-positive *S. aureus* and *Bacillus subtilis (B. subtilis)*. It was found that, regardless of the bacterial strain, only nano-topographic features present less density of attached bacteria (Figure 3).

This study shows that, in what concerns topographic cues when the surface presents attractive chemistry, higher surface roughness parameters increase the probability of bacterial colonization and biofilm formation. This fact is a consequence of the similarity between the morphology/topography of the extracellular matrix and nano and microroughness in opposition to surfaces with only nanotopographic profile.

Moreover, another problem arises as determining surface topography importance is still not accurate, considering the complex interactions between bacteria and surfaces [26,27].

### 2.2. Chemical Action

In opposition to the number of works reported in the literature regarding the influence of the topographic cues of polymeric coatings on the microbial activity, when the subject is focused on the chemistry of the surface, a significant number of published research is available.

Hybrid polymeric/metal coatings have been widely reported in the late years. Usually, these coatings include metal ions or nanoparticles, which are impregnated in the coating for further release or not to conduct the antimicrobial activity [28]. Silver is by far the most studied metal in such approaches since its antimicrobial properties are well established for a wide range of microorganisms [29].

**Figure 4:**
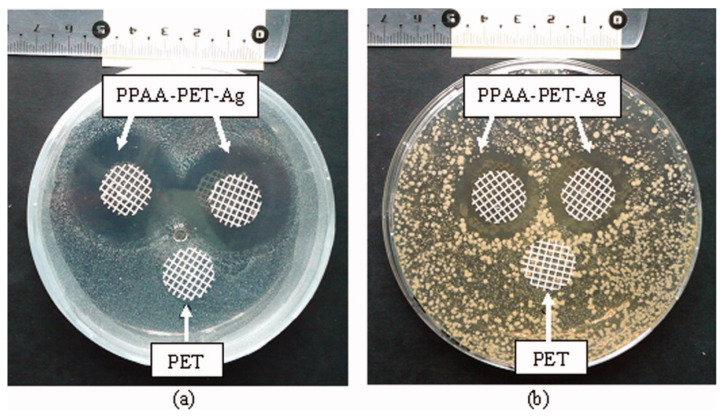
Antibacterial analysis of Ag loaded and control PET meshes: (**a**) *S. aureus*; (**b**) *E. coli*. Reprinted from [31], with permission from Elsevier.

In a study conducted by Pishbin et al., Ag NPs were encapsulated into a composite formulation of chitosan and bioactive glass to coat 316 stainless steel samples, by a single-step electrophoretic deposition (EPD), for orthopedic applications [30]. Besides helping to avoid the initial burst release, the encapsulation of the nanoparticles also hinders possible environmental hazards associated with the release of Ag NP. However, although the coatings proved to be efficient towards *S. aureus* up to 10 days, the concentration of Ag NPs revealed to be cytotoxic to osteoblast-like cells up to 7 days [30]. Further studies are proposed in order to refine the correct concentration of the Ag NP. In a different approach, the antimicrobial ability of PET coated and uncoated surfaces was evaluated against bacteria. Herein, PET meshes were coated with a polyacrylic acid/Ag NPs formulation by a plasma polymerization technique [31]. Before the coating procedure, the Ag NPs were entrapped into the polyacrylic acid polymeric network. After 24 h, the coated meshes presented a visible inhibition halo towards *S. aureus* and *E. coli* compared to the uncoated PET mesh (Figure 4).

No bacterial growth reduction was observed for the uncoated meshes. Another approach describes the preparation of a polyacrylate-based hydrogel with Ag NPs to coat titanium implants, by electrosyntherization [32]. The antimicrobial activity of the coatings was tested using the most common pathogens in orthopedic infections. Besides, the interaction with osteoblasts was also assessed. The results show that tuning of the Ag ion release was conducted properly since the coatings were able to maintain the antimicrobial activity without causing any harm or negative response to the osteoblasts present at the implant interface. The release of Ag NPs caused some concerns about their possible toxic behavior towards the cell, unlike Ag ions [33].

Following this route, a research team proposed a coating production that allows the release of Ag ions while maintaining the Ag NPs attached to the network to reduce toxicity [34]. The stabilization of the Ag NPs was achieved by their incorporation into a poly(butyl acrylatemethyl methacrylate) copolymer. This formulation was then used to coat glass slides by a dip coat technique. After six days, the coatings proved to maintain the Ag NPs while releasing silver ions to the surrounding media. To coat Ti screws for biomedical applications, silver nanoparticles were embedded into a PP-g-PEG graft copolymer [35]. After the coating formulations were complete, the Ti screws were dipped into the solution and dried for 5 min. This last step was repeated 20 times until a film was covering the screw. The antimicrobial effect was determined by the exposure to *S. aureus*, showing promising results. After 21 days of implantation, the screws were able to maintain Ag NPs attached to their surface showing that the coating was resistant to the tapping forces to which the screws were susceptible.

Using a sol-cast method, new chitosan/Ag/ZnO composite films were prepared for coating glass slides [36]. After the Ag NPs impregnation, it was possible to observe that the particles presented a granular and spherical morphology and were uniformly distributed along the composite film. In what concerns the antimicrobial tests, a wide range of bacteria was used. After the tests, it was possible to conclude that the chitosan/Ag/ZnO coatings presented higher antimicrobial activity than chitosan/ZnO and chitosan/Ag films. This fact proves that the antimicrobial effect is enhanced by incorporating Ag and ZnO together within the chitosan polymeric network [36]. The initial color of chitosan is not significantly affected by the incorporation of Ag and ZnO.

**Figure 5:**
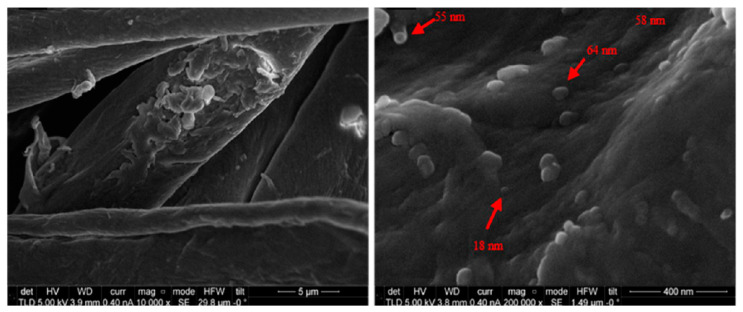
SEM micrographs of cotton fabric coating with ZnO/chitosan coating. In the right image the arrows show the size of the ZnO nanoparticles. Reprinted with permission from [37]. Copyright 2016 American Chemical Society.

To avoid the use of silver nanoparticles, Tzanov et al. described the preparation of hybrid chitosan/ZnO coatings for cotton fabrics [37]. In this work, the deposition of both ZnO nanoaprticles and chitosan was performed by a sonochemical technique. The ZnO concentration in the film and ultrasound irradiation time (processing time) were varied to achieve the highest antibacterial effectiveness without being cytotoxic. Figure 5 displays the morphology of the cotton fabric coated with ZnO/chitosan coatings.

In what concerns the antimicrobial activity of the coated cotton fabric, the most effective coating was achieved for 30 min deposition time and 2 mM concentration of ZnO NPs. The incorporation of chitosan enhanced the final biocompatibility of the coatings avoiding the possibility for them to become harmful for human health. Concomitantly, the coatings containing chitosan displayed superior antimicrobial potential against *S. aureus* and *E. coli*. Hospital laundry conditions were simulated by multiple washing cycles at 75 °C to assess its influence on the antimicrobial properties of the coatings. Results show that chitosan improved the durability of the antimicrobial effect. The final cytotoxicity of the coating was also positively influenced by the incorporation of chitosan since chitosan/ZnO coatings presented higher fibroblast viability in comparison with ZnO coatings.

Composite Ag NPs/alginate coatings were applied to the cotton fabric to study the antibacterial activity of the coated fabric throughout successive washes [38]. In the coating formulation, alginate plays two roles, as it is concomitantly the reducing and stabilizer agent of the metal nanoparticles. The coating procedure consisted of the immersion of the fabrics into the coating mixture, following by padding, drying at 75 °C for 15 min, and lastly, the curing process at 120 °C for 3 min. Against *E. coli*, *S. aureus*, and *P. aeruginosa*, the coated fabrics demonstrated promising antibacterial activity. However, with successive washing, the antibacterial potential suffered a slight decrease [38].

**Figure 6:**
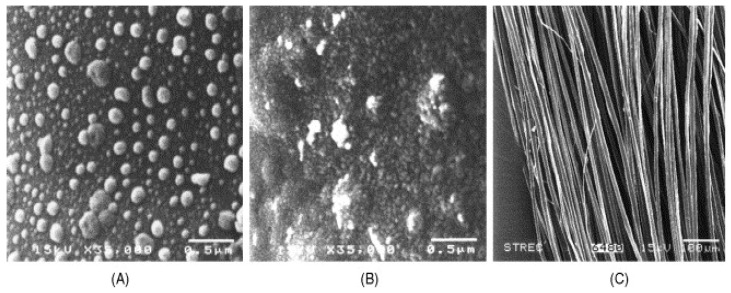
SEM micrographies of fibers coated with 20 cycles of dipping into PDADMAC and PMA capped Ag NPs: (**A**) nylon; (**B**) and (**C)** silk. Reprinted from [39], with permission from Elsevier.

Another study reports the coatings of nylon and silk fibers by a layer-by-layer dipping technique. Multilayers of poly (diallyldimethylammonium chloride) (PDADMAC) and silver nanoparticles pre-capped in poly (methacrylic acid) (PMA) constituted the final fiber coating [39]. Figure 6 shows the fibers after 20 cycles of dipping.

After coating solution preparation and dipping of the nylon and silk fibers, the antimicrobial potential was assessed using *S. aureus*. Results show that there was ca. 80% bacteria reduction on coated silk fibers and around 50% reduction for the coated nylon fibers. Even though these coatings seem to be more effective for silk fibers, it is suggested that this technique could be useful for the design and manufacture of both synthetic and natural fibers with antimicrobial properties.

**Figure 7:**
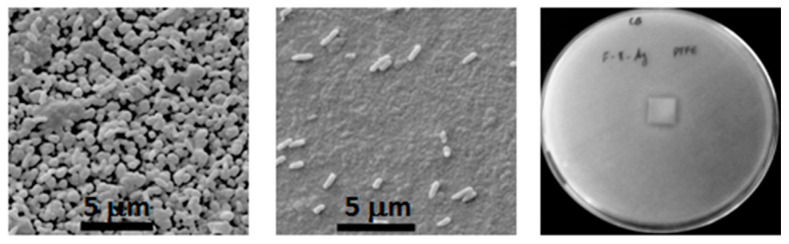
SEM images of PTFE/PA coating with *P. aeruginosa* (**left**); PTFE/PA with silver (**middle**); inhibition halo formed for PTFE/PA with silver (**right**). Reprinted from [40], with permission from Elsevier.

Using a different approach, polytetrafluorethylene (PTFE)/polyamide (PA)/Ag composite coatings, produced by PVD, were deposited onto PTFE substrates [40]. In this work, the coatings were tested against one of the most hazardous microorganisms responsible for nosocomial infections: *P. aeruginosa*. The incorporation of PA in the coating formulation helped to decrease the hydrophobicity of the PTFE substrates. Besides, when Ag was added, the surface of the coating assumed a negative value of zeta potential and a high polar component of the surface energy. With the release of Ag ions, the coating acquired antimicrobial properties, which proved to be effective against *P. aeruginosa* (Figure 7).

Multilayer thin films made from a mixture of poly(allylamine hydrochloride) (PAH), poly(acrylic acid) (PAA) and polyacrylamide (PAAm), (PAH/PAA/PAAm), with Ag nanoparticles were synthetize to coat planar surfaces and magnetic colloidal particles to evaluate the antibacterial effect [41]. The influence of the thickness of the entire film and the concentration of Ag nanoparticles in the antibacterial properties was also assessed. Results suggest that the inhibition halo increases with the increase of Ag concentration in the film. In addition, the release of silver into the surrounding media occurs by oxidation of the surface of the nanoparticles. The magnetic microspheres coated with the described films were suggested as possible antibacterial deliver agents towards precise locations.

Besides silver, copper nanoparticles have also been studied for antimicrobial purposes. The possibility to coat stainless steel devices with a poly(ethylene glycol diacrylate)/copper-based nanoparticles hydrogel was described elsewhere [42]. The coatings were attached to the substrates by fast and straightforward electrochemical polymerization. Antimicrobial tests using *S. aureus* and *E. coli* demonstrated the ability of the CuNPs-PEGDA coatings to inhibit the growth of the studied bacteria.

**Figure 8:**
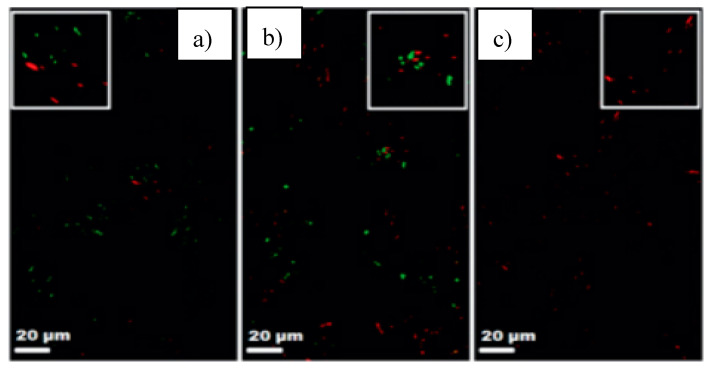
Live and dead bacteria on Cu-PES samples: (**a**) t = 0; (**b**) t = 30 min; (**c**) t = 60 min (low intensity light irradiation). Reprinted from [43], with permission from De Gruyter.

More recently, the conjugation of polymeric materials and copper has been studied for antimicrobial purposes using the metal nanoparticle approach and other processing techniques. An example of that is the work conducted by Rtimi et al. where Cu-polyester (Cu-PES) coatings are prepared by a sputtering technique [43]. Herein, the coatings could inactivate *E. coli* within 45 min in both anaerobic and aerobic media (Figure 8). The inactivation occurred not only under low intensity visible light but also for dark conditions.

Aside from incorporating metal ions or nanoparticles, there are studies where antimicrobial and antibacterial coatings were prepared only using polymeric materials. Due to its biocidal properties [44], N,N-dodecylmethyl-polyethylenimine (PEI) received special attention and was included in a series of studies. In a work conducted by Park et al., glass and polyethylene slides were dipped into PEI-based hydrophobic solutions [45]. After the dipping process, the solvent was removed by evaporation. The coated samples were then incubated with *S. aureus* and *E.coli*. Results show that the coated slides were able to kill bacteria, probably due to the rupture of the cellular membranes.

Klibanov and co-workers published several works using PEI, where both formulation and coating procedures were evaluated. The use of linear and branched PEI and other PEI derivatives as a paint-like coating in glass slides was also reported [46,47]. The antibacterial action was validated by evaluating the antibacterial activity of the coatings in contact with *S. aureus* and *E. coli*.

In one of the most recent published studies, PEI was used to coat glass slides using a painting technique. As a result, coatings proved to be lethal to both *S. aureus* and *E. coli* [46]. Following the paint-like coating approach, most recently, organo-soluble quaternary chitin polymers were synthesized to create bactericidal paint [48]. The immobilization of the prepared polycationic paint was achieved without covalent bonding. The coatings proved to be effective against drug-sensitive and drug-resistant bacteria. When in contact with the bacteria, the cationic polymers can break their cell membrane, causing it to leach out its intracellular constituents. As a consequence of the leaching, the bacterial cell dies.

**Figure 9:**
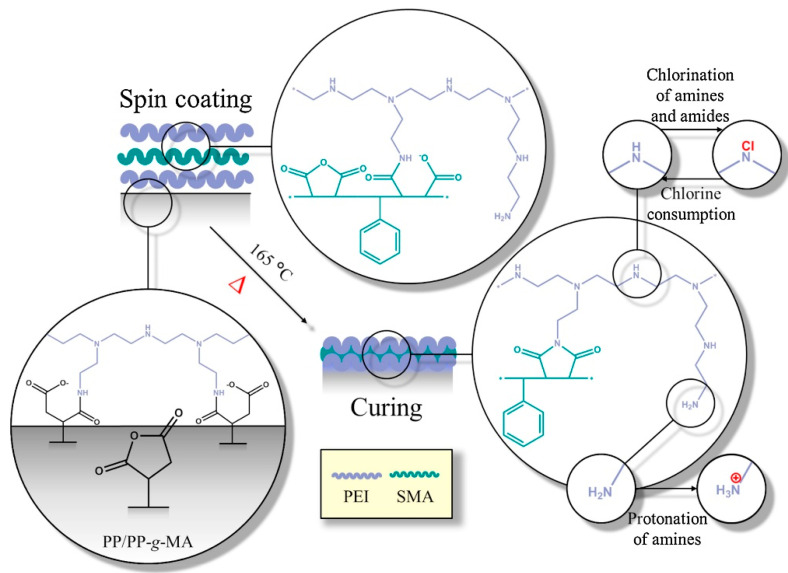
Schematic representation of the preparation of the PEI/SMA coating. Reprinted from [49], with permission from Elsevier.

The preparation of a self-healing antimicrobial polymeric coatings is reported in the literature. In a work, using a spin coating technique, multilayers of PEI and styrene maleic anhydride copolymer (SMA) were deposited onto PP-based substrates and then cured, as shown in Figure 9 [49]. Due to the composition of the coating, it was possible to tune several properties. The hydrophobic character was conferred by the styrene subunits, while the antimicrobial potential was related to the cationic primary amine groups and chlorination of N-halamine formed groups. As a result, the coatings maintained their antimicrobial activity even when increasing the organic load conditions of *E. coli*. In addition, the coatings proved to have self-healing properties, after exposure to both acid and alkaline surrounding media, by being able to restore their characteristic chemical bonds under heat [49].

**Figure 10:**
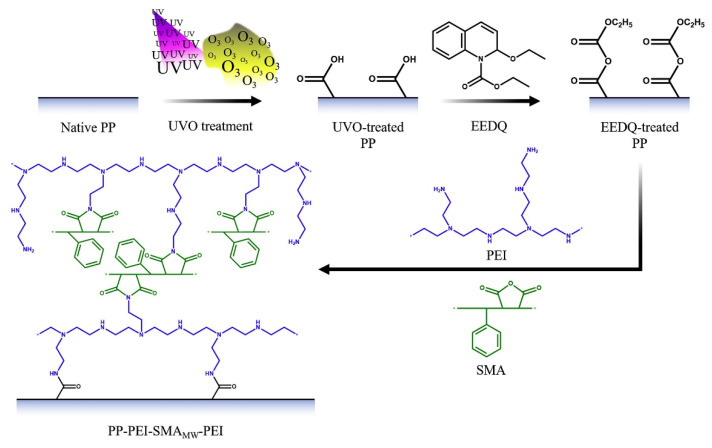
Representation of the activation of PP substrates and further coating. Reprinted from [50], with permission from Elsevier.

The same group investigated the antimicrobial potential of PEI/SMA multilayer coatings on PP based substrates with the variation of SMA molecular weight [50]. Furthermore, the storage stability was also evaluated. Before coating, the PP substrates were activated in order to enhance the binding of the coating. Square substrates were exposed to UV-Ozone irradiation (UVO) to promote the formation of carboxylic acid groups by a photo-oxidation reaction. Then, to create anhydride groups on its surface, the substrates reacted with 2-ethoxy-1-ethoxycarbonyl-1,2-dihydroquinoline (EEDQ). This step intended to turn the surface of the PP substrate more reactive when in contact with the primary amine groups of PEI. The activation and coating of PP substrates are represented in Figure 10.

Results show that the molecular weight of SMA influenced the surface energy of the coating [49]. With the increase of the SMA molecular weight, the surface energy decreased. Among the different molecular weights studied, the SMA with the lowest molecular weight (6 kDa) originated films with enhanced biocidal activity against *E. coli*.

In a different approach, using electrochemical deposition, coatings of polypyrrole (PPy) and PPy/PEG were deposition on novel titanium allow electrode [51]. This work proved that the composite coatings were more effective against *E. coli* than the single PPy coating. Besides, the surface characteristics of the composite PPY/PEG coating seem to be the critical factor in the inhibition of *E. coli*.

**Figure 11:**
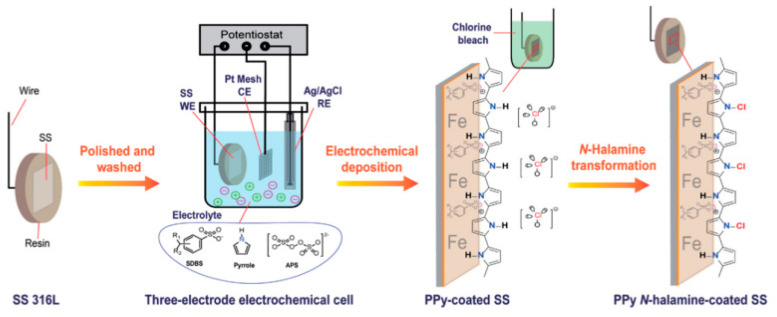
N-halamine based PPy coating preparation scheme [52]. Reprinted from Engineered Science Publisher.

The preparation of PPy coatings with both antimicrobial and electrical properties was suggested elsewhere [52]. PPy was deposited on stainless steel substrates by an electrodeposition technique. However, to activate the antimicrobial effect of PPy, after the deposition, the prepared coating was treated with chlorine bleach. As a result, the final coating was a N-halamine based PPy (Figure 11).

When in contact with *S. aureus*, the prepared coating could inactive the bacteria after 60 s for more than one test cycle. Therefore, it can be concluded that the coating effectiveness was not affected by a first cycle in contact with the bacteria. Multifunctional coatings for protecting steels in harsh environments were examples of applications suggested for the prepared coatings [52].

**Figure 12:**
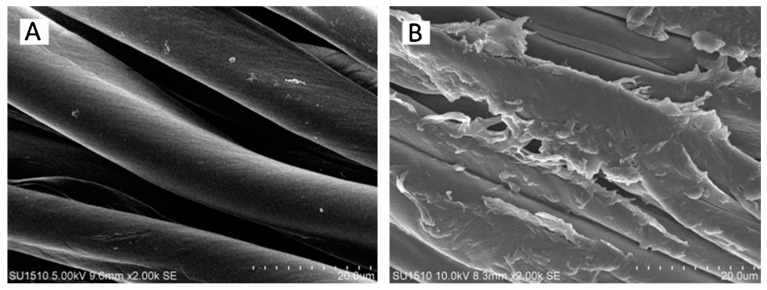
SEM images of: (**A**) uncoated cotton fabric, (**B**) coated cotton fabric. Reprinted from [53], with permission from Elsevier.

As aforementioned, besides flat samples, other materials with different geometries, like fabrics, have also been tested with different coatings to increase their antimicrobial action. In this sense, an innovative N-halamine chitosan derivative was synthesized and used to coat cotton fabric [53]. By dipping the fabric into the polymeric solution, complete coverage of the fabric was achieved (Figure 12).

The coated fabrics show to be able to inactive both *S. aureus* and *E. coli* within a contact time of 5 min. CVD deposition technique was used to create polymeric coatings on nylon fabric substrates [54]. Coatings up to 540 mg/cm^2^ of poly(dimethylaminomethyl styrene) were produced without affecting the color or the feel of the nylon fabric. No leaching from the fabric was observed. Following the ASTM E2149-01 standard, the coatings were tested for antimicrobial activity using *E. coli* and *B. subtilis*. Coatings with different concentrations were found to be very effective against both studied bacteria.

Especially during the last decade, the study of innovative antimicrobial coatings acquired new routes of investigation base on multifunctional coating preparation and deposition. In a study conducted by Ming et al., polymeric coatings with both antifogging and antimicrobial function were synthetized, leading to semi-interpenetrating polymer networks (SIPN) [55]. These structures were constituted by quaternized poly(2-(dimethylamino)-ethyl methacrylate-co-methyl methacrylate) and polymerized ethylene glycol dimethacrylate. After the coating solution preparation, glass slides were coated by a spin coating technique. The dual functionality of the coatings was evaluated, and results show that the antifogging behavior was related to the hydrophilic/hydrophobic balance, while the antimicrobial effect derived from the hydrophobic quaternary ammonium compound, which was covalently bonded. The coatings proved to be effective in killing both Gram-positive *Staphylococcus epidermidis* (*S. epidermidis*) and Gram-negative *E. coli*. Moreover, it was concluded that the bacteria killing mechanism of the coatings is based on contact killing [55]. No leaching of bactericidal species was observed.

## 3. Conclusions and Future Perspectives

From the presented bibliographic review, it can be concluded that the published work that reports only the topographic cues as the cause of the inhibition of bacterial adhesion and spread are few, and very often, contradictory results are described between different manuscripts. Usually, the topographic cues are reported along with the chemical action of materials that constitute the coatings. The significant percentages of work report the use of different chemical substances with the emphasis in the use of metals, such as silver, that confer to the polymeric surfaces the desired antimicrobial properties.

Although this path can, in the short term, be presented as a solution in some very specific applications, several problems can arise from these approaches. Firstly, it is very controversial the optimal concentration of metallic material that can, simultaneously, present antimicrobial properties and provoke no harmful consequence to eukaryotic cells. Only very few works report in vitro studies with both types of cells to conclude about the true efficacy of the developed surfaces. Secondly, researchers in the field of genomics conclude that the metabolic pathways that induce metabolic resistance of the microorganisms towards antibiotics are the same that allow the microorganisms to develop resistance to metals. Therefore, it is only a matter of a short time before the approach considering metallic elements as antimicrobial agents is surpassed.

Therefore, the authors believe that it is of the most urgency to begin to explore new paths for the development of new strategies for antimicrobial surfaces other than the incorporation of metallic (nano)particles.
